# Laparotomic Myomectomy in the 16th Week of Pregnancy: A Case Report

**DOI:** 10.1155/2014/154347

**Published:** 2014-03-04

**Authors:** Lavinia Domenici, Violante Di Donato, Maria Luisa Gasparri, Francesca Lecce, Jlenia Caccetta, Pierluigi Benedetti Panici

**Affiliations:** Department of Obstetrics, Gynecology and Urologic Sciences, “Sapienza” University, Viale Del Policlinico 155, 00155 Rome, Italy

## Abstract

Myomectomy is rarely performed during an ongoing pregnancy because of fear of miscarriage and the risk of an uncontrolled haemorrhage necessitating a hysterectomy. In cases where myomectomy is undertaken, most are performed at the time of cesarean section or with a laparoscopic approach. We report a case of a successful laparotomic myomectomy in the 16th week of pregnancy. A 35-year-old primigravida was admitted to our department with acute abdominal pain and hydronephrosis (serum creatinine 1.6 mg/dL). Imaging revealed a large implant myoma compressing the bladder, ureters, rectus, and gestational chamber and causing hydronephrosis. Laparotomic myomectomy was successfully performed and pregnancy continued uneventfully until the 38th week when a cesarean section was performed. Surgical management of myomas during pregnancy is worth evaluating in well-selected and highly symptomatic cases.

## 1. Introduction

The estimated prevalence of uterine myomas during pregnancy varies from 0.3 to 15% [1]. Most uterine myomas remain asymptomatic during pregnancy but may result in obstetrical complications in about 10% of cases depending on their size, location, and number [2–4]. Pain is the main symptom reported in pregnancies with uterine myoma; however, in 2% of patients conservative medical therapy fails. In extreme cases some authors have advocated the interruption of pregnancy to relieve pain [5]. Myomectomy is generally avoided during pregnancy due to the high risk of haemorrhagic or obstetrical complications and no clear unanimous consensus exists, with a surgical approach reserved for cases of intractable abdominal pain and degeneration or rapid growth of myoma [6, 7]. Only a few cases of myomectomy in pregnancy have been reported in the literature [6, 8–10]. In this paper we report a case of myomectomy of subserous myoma with large base of implant causing hydronephrosis in the 16th week of pregnancy.

## 2. Case Presentation

**Figure 1:**
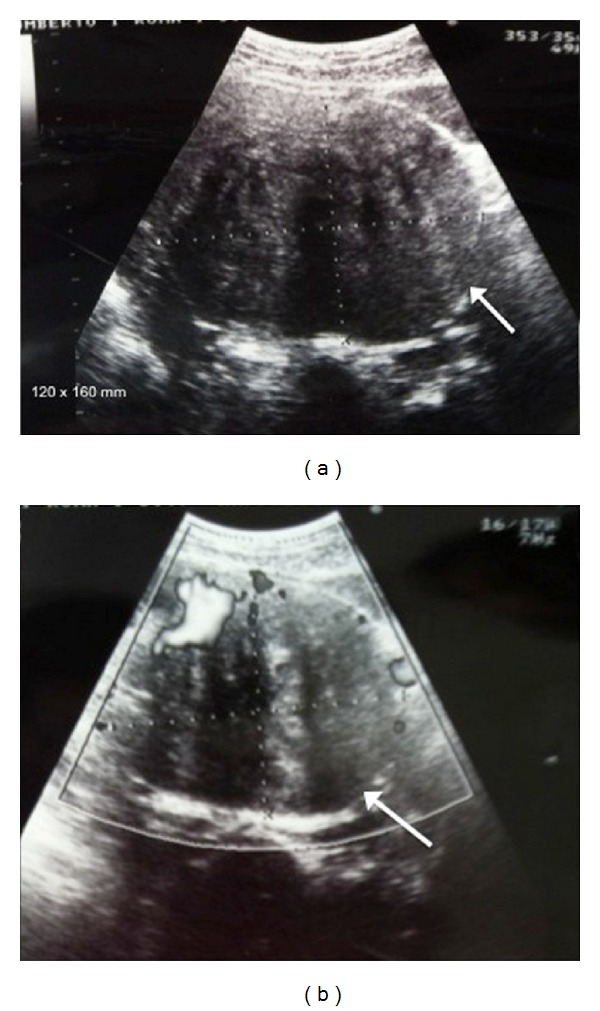
Myoma at ultrasonographic exam.

In January 2013, a 35-year-old primigravid Caucasian woman (BMI: 22) was referred to our university hospital in the 16th week of gestation for intractable pelvic pain. The medical history was uneventful. The patient reported a sense of pelvic heaviness, changes in urinary habits, lower abdominal discomfort, and unexplained back pain that had worsened over time. She had a normal white blood cell count but an increased value of serum creatinine (1.6 mg/dL). Obstetrical examination showed a large mass at the level of the posterior fornix. Abdominal ultrasound confirmed a viable fetus and a subserous large implant myoma (diameter of 20 cm; Figure 1), a mild bilateral dilatation of the renal pelvis (2 cm on the right-hand side and 3.5 cm on the left), and extrinsic compression of the low ureter with involvement of the initial portion of the ureters.

**Figure 2:**
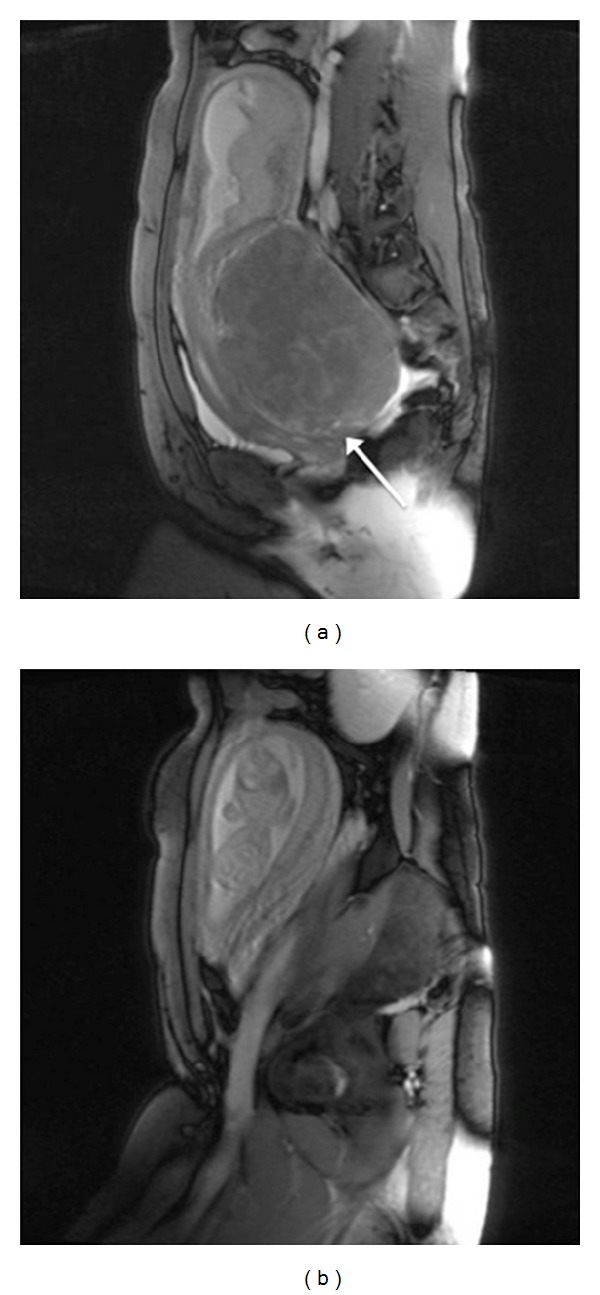
Bulky leiomyoma occupying the pouch of Douglas with signs of compression of ureters, bladder, sigma-rectus, and the gestational chamber; the presence of bulky leiomyoma (20 × 18 cm) with large base of implant (12 cm) occupying the pouch of Douglas. MRI also showed other multiple nodes, of which the bigger was of diameter 4 cm.

Magnetic resonance imaging (MRI) confirmed the diagnosis (Figure 2).

**Figure 3:**
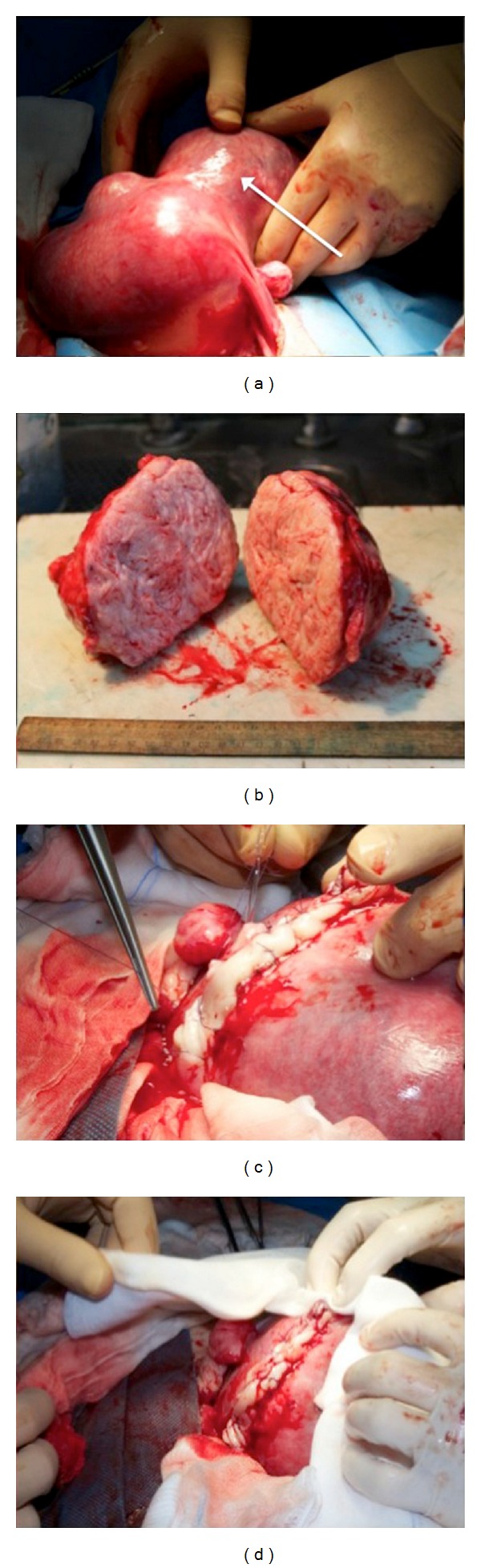
Myomectomy and uterine wall reconstruction.

Considering the increase in symptoms that were nonresponsive to analgesic therapy, due to organ compression and the level of serum creatinine, after extensive counselling, a myomectomy was planned. Given the volume and the location of the myoma and in order to be able to manage possible complications, laparotomic incision was chosen. After accurate operative field exposure, the huge myoma was removed (Figure 3). Reconstruction of the uterine wall was carried out using a two-layer monofilament absorbable 2.0 suture (Poliglecaprone 25). Other small fibroids were observed but not removed. Estimated blood loss and operation time were 250 cc and 90 minutes, respectively, and no intra- and postoperative complications occurred. An ultrasonographic control of fetal outcome was carried out 7 days after surgery which proved normal. Antibiotics (ampicillin plus sulbactam 3 gr for three times a day) and low molecular heparin were administered for 5 and 10 days, respectively. The patient was dismissed on the 5th postoperative day. Serum creatinine at discharge was 1.1 mg/dL.

Hydroxyprogesterone Caproate (341 mg/2 mL im) was dispensed for 15 days to prevent a possible miscarriage.

The patient was closely followed up with ultrasound and physical examination every four weeks until 24 weeks of gestation, every three weeks from 24 weeks to 34 weeks of gestation, and then every two weeks. Physiological fetal growth and an uneventful antenatal period were reported until 38 weeks of gestation when a cesarean section was performed.

The patient delivered a healthy female baby weighing 3250 gr with Apgar scores of 8 and 9 at one and five minutes, respectively. The maternal haemoglobin level, two days after caesarean section, was 12.2 g/dL. Mother and baby were discharged from the hospital after three days. The 6-week postnatal visit was within the norm.

## 3. Discussion

Laparotomic myomectomy is generally avoided during an ongoing pregnancy due to higher miscarriage and haemorrhage rates. Most cases are usually performed during a caesarean section at the end of the pregnancy.

In a large study of more than 6300 pregnant women, Coronado et al. reported a 1.9 times greater (95% CI, 1.6 to 2.2) incidence of complications in women with myomas compared with women without myomas [11].

Preterm delivery has been reported in approximately 15–20% of women with myomas, restriction of fetal growth in 10%, and malpresentation in 20% [2].

The increased risk of miscarriage was attributed to the increase in uterine contractions, degeneration, and growth of myoma.

The most common indication for myomectomy during pregnancy is acute severe abdominal pain that does not respond to analgesic therapy due to torsion of the subserous pedunculated myomas or rapid abnormal increase in myoma size, resulting in the compression and displacement of surrounding organs. It has been reported that if symptoms persist after 72 h of pharmacological therapy, surgical intervention must be considered [6, 12, 13].

An analysis of cases reported in the literature suggests that myomectomy during pregnancy can be considered safe. Studies have shown that women who undergo surgical intervention in the second trimester actually have better outcomes than those who opt for conservative management [10, 14] (Table 1).

In Lolis et al.'s study, of 13 patients who underwent myomectomy during pregnancy, only one miscarried, making a success rate of 92% [6].

The majority of interventions described in the literature are performed laparoscopically. Laparoscopy can be considered in selected cases (small, subserous pedunculated myomas). It is a valid option in the surgical management of pregnant women with symptomatic myomas, as it is less invasive and involves minimal postoperative pain and earlier postoperative ambulation [15].

Two cases are reported in the literature with large pedunculated myomas operated by the vaginal route [16].

In our case a laparotomic approach was chosen, because of the size (20 cm), the large base of implant, the location of myoma, and the acute syndrome of the patient.

Although a laparotomic approach for uterine myomas during pregnancy is rarely described, our experience suggests that it can be easily managed laparotomically by an experienced surgeon in selected cases, depending on the size, type, and position of the fibroids.

## 4. Conclusions

We believe that our experience provides reassurance for pregnant women with uterine myomas: the surgical management of uterine myomas during pregnancy can be successfully performed by expert surgeons on a case-by-case basis. Myomectomy during pregnancy should be performed only if unavoidable. In selected patients it could prevent miscarriage or an unacceptable obstetrical outcome. The surgical approach should be tailored to the patient and to the characteristics of the myoma. Clearly, an expert surgical and anesthesiological team is essential in order to reduce risk of complications.

Further investigation is needed to improve and better define the safety and feasibility of laparotomic myomectomy during pregnancy.

## Figures and Tables

**Table 1:** Case reports, case series, and a literature review.

Author	Patients number	Symptoms	Number of leiomyomas	Gestational age at treatment (week)	Weight of largest leiomyoma (g)	Week and type of delivery	Outcome (birth weight and Apgar score)
Michalas et al., 1995 [17]	1	Abdominal pain	1	15	NA	39	NA
Danzer et al., 2001 [18]	1	Abdominal pain and vaginal bleeding	1	12	NA	37 CS	A 3235 g—9/10 B 2810 g—9/10
de Carolis et al., 2001 [12]	18	Pain, fever, bleeding, 1 threatened miscarriage, 9 asymptomatic	1–4	6–24	NA	36–41 weeks 14 CS 2 VD	2550–3970 g 8/8–9/10
Lozza et al., 2011 [15]	1	Acute urinary retention	2	15 + 5	NA	35 + 5 CS	2280 g—9/9
Joó et al., 2001 [19]	1	Fetal postural deformity, oligohydramnios	1	25	NA	40 CS	3600 g—good
Celik et al., 2002 [14]	5	Abdominal pain	1–4	13–22	NA	CS	2800–3600 g—8–10
Hasbargen et al., 2002 [20]	1	Abdominal pain	1	18	1570	36 CS	2495 g—8/8
Umezurike and Feyi-Waboso, 2005 [21]	1	Abdominal pain	1	30	7700	38 VD	3500 g—8/10
Usifo et al., 2007 [22]	1	Abdominal pain, vomiting, diarrhoea	1	13	2000	38 CS	3990 g—good
Suwandinata et al., 2008 [4]	1	Abdominal pain	2	15	649	37 Cs	2950 g—8/9
Bhatla et al., 2009 [13]	1	Subacute intestinal obstruction	1	19 + 3	3900	38 VG	2740 g—good
Leite et al., 2010 [23]	1	Abdominal pain	1	17	NA	39 CS	3315 g—9/10
Isabu et al., 2010 [24]	1	Abdominal pain	1	14	NA	37 CS	2700 g—good
Leach et al., 2011 [5]	1	Pelvic pain, constipations, urine retention	2	11	NA	40 + 3 CS	4356 g—9/9
Doerga-Bachasing et al., 2012 [9]	1	Abdominal pain and vomiting	1	10	2745	36 CS	Normal—optimal
